# Online proprioception feeds plasticity of arm representation following tool-use in healthy aging

**DOI:** 10.1038/s41598-020-74455-5

**Published:** 2020-10-14

**Authors:** Salam Bahmad, Luke E. Miller, Minh Tu Pham, Richard Moreau, Romeo Salemme, Eric Koun, Alessandro Farnè, Alice C. Roy

**Affiliations:** 1 Laboratoire Dynamique du Langage, CNRS UMR 5596, University Lyon 2, Lyon, France; 2 Integrative Multisensory Perception Action & Cognition Team—ImpAct, Lyon Neuroscience Research Center CRNL INSERM U1028, CNRS UMR5292, University UCBL Lyon 1, Lyon, France; 3 University of Lyon, Lyon, France; 4 Laboratoire Ampère, CNRS UMR5005, INSA Lyon, Univ Lyon, 69621 Villeurbanne, France; 5 Hospices Civils de Lyon, Mouvement et Handicap & Neuro-immersion, Lyon, France; 6 Center for Mind/Brain Sciences (CIMeC), University of Trento, Rovereto, Italy; 7 Present Address: 16 Avenue du Doyen Jean Lépine, 69500 Bron, France

**Keywords:** Human behaviour, Cognitive ageing, Perception

## Abstract

Following tool-use, the kinematics of free-hand movements are altered. This modified kinematic pattern has been taken as a behavioral hallmark of the modification induced by tool-use on the effector representation. Proprioceptive inputs appear central in updating the estimated effector state. Here we questioned whether online proprioceptive modality that is accessed in real time, or offline, memory-based, proprioception is responsible for this update. Since normal aging affects offline proprioception only, we examined a group of 60 year-old adults for proprioceptive acuity and movement’s kinematics when grasping an object before and after tool-use. As a control, participants performed the same movements with a weight—equivalent to the tool—weight-attached to their wrist. Despite hampered offline proprioceptive acuity, 60 year-old participants exhibited the typical kinematic signature of tool incorporation: Namely, the latency of transport components peaks was longer and their amplitude reduced after tool-use. Instead, we observed no kinematic modifications in the control condition. In addition, online proprioception acuity correlated with tool incorporation, as indexed by the amount of kinematics changes observed after tool-use. Altogether, these findings point to the prominent role played by online proprioception in updating the body estimate for the motor control of tools.

## Introduction

Adapting the environment to our needs often requires the use of tools. Whether for leisure or for work, tools are present in many aspects of our life, but their skillful control represents a challenge for our sensorimotor system. Imagine a surgeon using tools with different sizes. The control exerted over the tip of different tools ought to be accurately adapted. To achieve this flexible control, the state of the effector (e.g., its position, dimensions, etc.) needs to be continuously monitored. Such body estimate (or schema) is a critical element of motor control: it provides the internal model with the information needed to execute actions correctly^1,2^. Furthermore, it can update this information if rapid changes of the effector occur, as when grabbing objects with tools that temporarily increase the size of the effector (i.e., arm + tool length). It is indeed well-established that using arm-elongating tools induces a temporary increase of the arm-length representation, as indexed by changes in movement kinematics and tactile perception^3–9^.

In the last decade, tool-use has emerged as a valuable paradigm to investigate plasticity of the body representation for action^6,10–12^. In a typical pre/post tool-use paradigm, participants are required to reach and grasp objects with their hand, before and after using a mechanical grabber that elongates their arm’s reaching capabilities. The pre-post tool-use comparison has consistently outlined a pattern of kinematics changes in the transport phase of the ensuing movements (noteworthy, without the tool), displaying smaller peaks (of acceleration, velocity and deceleration) and longer latencies (of the same peaks). This pattern has been taken as the kinematic signature of the so-called tool incorporation into the body state estimate. Indeed, movements performed after tool-use are akin to those observed when reaching with a naturally longer arm^6,13^. Not least, the kinematic pattern generalizes to free hand movements that were not specifically exposed to tool-use (e.g., pointing instead of grasping^6^), as well as to positions not exposed to tool-use (e.g., orthogonal to tool-use^13^).

Recent studies showed that both vision and proprioception contribute to updating the arm estimate following tool-use^8,13^. Most importantly, proprioception is both sufficient and necessary to trigger such a plasticity, as proprioceptive deafferentation prevents updating the metrics of the arm estimate guiding our action^14^. Overall, these findings point toward a critical role played by proprioception in lengthening the estimated metrics of the arm, which would thus influence subsequent movements execution, when the tool is no longer held^11^.

This raises the question of whether online, or memory-based proprioception is responsible for the changes in free hand kinematics following tool-use. While the need for a constant monitoring of the effector state would favor the online access hypothesis, an alternative possibility is that stored information is used, as tool-use effects are observable while the tool is no longer held. Proprioceptive information may indeed be accessed in real time (online) or, conversely, stored in order to be accessed later on (memory-based). This is reflected in different tasks for testing position sense. In the contralateral matching task, a joint displacement is experienced and the participant has to reproduce the joint angle with his/her opposite arm. In the ipsilateral matching task, the participant experiences the joint displacement only for a few seconds, then his/her arm is put back in the starting position and he/she has to reproduce the joint angle with the same arm. While the contralateral task typically taxes the online access to proprioceptive information, the ipsilateral task taxes the offline access to proprioception^15^. Interestingly, normal aging is known to affect proprioception^15,16^, but it does not affect it evenly. Old adults are reported with a deficit regarding mostly the offline, as compared to the online access to proprioceptive inputs^17,18^.

Here we leveraged this dissociation to test whether tool-use effects on kinematics are mediated by online or offline proprioception processes. To this aim, we first assessed upper limb proprioceptive sensitivity in young adults (average 25 year-old), as well as in group of old adults (average 60 year-old) with both ipsilateral and contralateral matching tasks. Several factors are at play in position matching tasks, as people may code for both joint position and kinaesthetic amplitude of the movements (see^19^). For the sake of comparison, here we leveraged tasks that already documented the off-line/online dissociation in old adults^17,18^. Based on previous work^17^ we predicted that old participants would report less accurate proprioception in the ipsilateral, but not in the contralateral matching task. Then, we assessed the same group of old participants for the emergence of the changes in movement’s kinematics that have been typically reported after tool-use^11^. In line with the hypothesis that efficient motor control requires online monitoring of the effector state estimate, we predicted that typical kinematic signature of tool-use should be observed in old adults with impaired offline, but spared online access to proprioception.

## Methods

### Participants

Twenty participants (six males and fourteen females, mean age = 60.1 ± 5.05, range 51–69) took part in this study. Three participants were left-handed as assessed through the Edinburgh Handedness Inventory^20^. None of the participants reported neurological or psychiatric disorders nor peripheral vascular disease or peripheral neuropathy. All participants had a normal or corrected-to-normal vision and audition and a normal range of upper limb movements. Participants provided written informed consent prior taking part in the study, which was approved by the French ethics committee (Comité de Protection des Personnes Sud-Est IV). All experimental procedures conformed to the Helsinki declaration^21^.

### Experimental design

The experiment consisted of two sessions run over two consecutive days. A cognitive and proprioceptive assessment were performed on the first day, together with the first experimental session that could be the tool-use session or the weighted-wrist session. The second day was an experimental session only (tool-use or weighted-wrist), the order of sessions being counterbalanced across participants. As in previous work using this paradigm^6^, the weighted-wrist session served as a within-subject control condition aimed at ruling out any unspecific fatigue effects due to the weight of the tool, or test–retest effects, on arm representation. For this reason, the weight attached to the wrist in the weighted-wrist session equaled the weight of the tool. If fatigue or repeated testing affects arm kinematics, this would be captured by this control session.

#### Cognitive and proprioceptive assessment

We assessed participants global cognitive function by administering the Mini Mental State Examination (MMSE) test^22^. The mean score value was 27.5, ranging from 24 to 30, thus highlighting no cognitive impairment in any of our old adult participants. In addition, a selection of five subtests of the Rivermead Assessment of Somatosensory Performance (RASP)^23^ was used to exclude any obvious somatosensory impairment. The mean score value was 98.3%, ranging from 87.5 to 100% of correct answers, confirming the absence of pathological impairment.

Further, we assessed online and offline proprioception through the *joint position matching task* performed in remembered and concurrent tasks^24^. As there is no standardized scale, or cut-off to define proprioceptive impairment, we additionally enrolled and tested both offline and online proprioception in a group of 20 young healthy adults (ten males and ten females, mean age = 25.4 ± 3.2, range 21–35). Young participants completed the proprioceptive assessment only. This allowed us to assess whether our old participants’ population was indeed impaired as compared to young participants. They also provided written informed consent prior taking part in the study, which was approved by the French ethics committee (Comité de Protection des Personnes Sud-Est IV), and all experimental procedures conformed to the Helsinki declaration.

We used a custom made apparatus similar to the one of Allen et al.^25^. In this setup, participants are blindfolded and their forearms are strapped to lightweight paddles aligned with the elbow joint. An inclinometer attached to each paddle directly displayed the value of the angular flexion of the elbow (precision value = 0.1°).

In the ipsilateral remembered matching task, an experimenter raised the paddle supporting the participant’s forearm to a target angle (20° or 40° elbow flexion from the initial position) in the sagittal plane, maintained this position for 5 s as assessed by a stopwatch, and returned the arm to its initial position. The participant was then required to reproduce the position with the same forearm, with the use of proprioceptive memory. The experimenter maintained a steady pace for each trial. This task was done similarly for both arms: Ipsilateral Remembered Right (IRR) and Ipsilateral Remembered Left (IRL).

In the Contralateral Concurrent Matching task (CCM), a similar procedure was undertaken involving the passive displacement of the right forearm to a target angle (as before 20° and 40° elbow flexion from the initial position). In this task, the dominant forearm was set and maintained in the target position while the participant performed the matching with his/her non-dominant forearm.

In all tasks alike, each target angle was administered 6 times in a randomized order for a total of 12 trials per task. We used the absolute matching error value, as a measurement of proprioceptive acuity to compare both groups^16^. For the three tasks, the absolute error value on every trial was computed as the unsigned difference between the reproduced/matched angular position and the reference one (20°/40°). The error values units were in angular degrees.

#### Tool-use and weighted-wrist sessions

The two sessions each consisted in three phases: a pre- and post-phase separated by a tool-use or a weighted-wrist phase, all performed in a dimly illuminated and sound-attenuated room. Participants were comfortably seated in front of a table, on an adjustable chair, their dominant hand closed in a pinch-shaped grip on a starting switch. They had to reach and grasp a wooden block (10 × 2.5 × 5 cm, weighing 96 g) placed on the table at a distance of 35 cm along the sagittal axis, in line with participants’ right shoulder (or left shoulder for left-handed participants). Importantly, as in previous studies using the same paradigm^4,6,13,26,27^, the object was always located inside the arm’s reaching space, thus preventing potential confounding effects of tool-use on space representation.

### Pre and post phases

**Figure 1:**
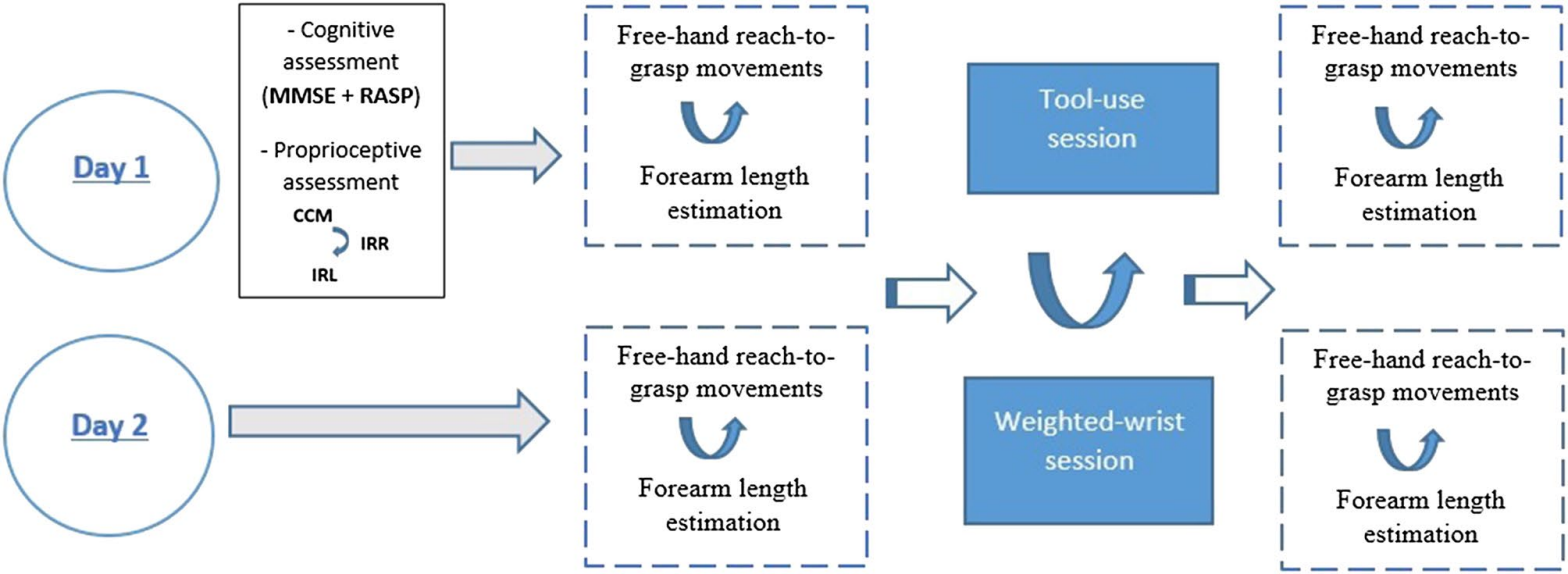
Within subject design run over two consecutive days: the first day consisted of cognitive and proprioceptive assessments followed by either a tool-use session or a weighted-wrist session. Curved blue arrows indicate the counterbalancing of session (tool-use and weighted-wrist) and the counterbalancing of pre-post tasks (free-hand reach-to-grasp movements and forearm length estimation) between subjects.

In the pre and post phases, participants performed two tasks: a free-hand reach-to-grasp task and a forearm length estimation task. While the former informs about unconscious changes in arm body estimate via kinematics, the latter informs about subjective, conscious changes in arm length representation^13^. Free-hand reaching movements and forearm estimation tasks were proposed in a counterbalanced order across participants (Fig. 1).

In the free-hand reach-to-grasp task, participants had to reach, grasp, and lift the object with their dominant hand for 18 trials. At the beginning of a trial, participants were asked to keep their dominant hand in the starting pinch-grip position, i.e. the tips of the thumb and the index in contact, pressing down the starting switch located in the proximal edge of the table, facing the object. After a random delay (ranging between 1 and 2 s), an auditory ‘go’ signal was produced and subjects had to reach, grasp and then raise the object keeping it between their thumb and index fingers. Then they had to put the object back on the table and return to their starting position.

In the forearm length estimation task (18 trials), blindfolded participants were asked to estimate the length of their dominant forearm from the elbow to the wrist. To that aim, starting from the switch, upon the auditory ‘go’ signal they had to slide their dominant index finger horizontally on the table (following a fronto-parallel axis) for a distance corresponding to the estimation of their forearm length.

To remind participants of the forearm distance to reproduce, the experimenter named and delivered a tactile stimulation on her dominant wrist and elbow before the first trial. Once a trial performed, participants had to return to the starting position and wait to perform the next trial.

### Tool-use phase

**Figure 2:**
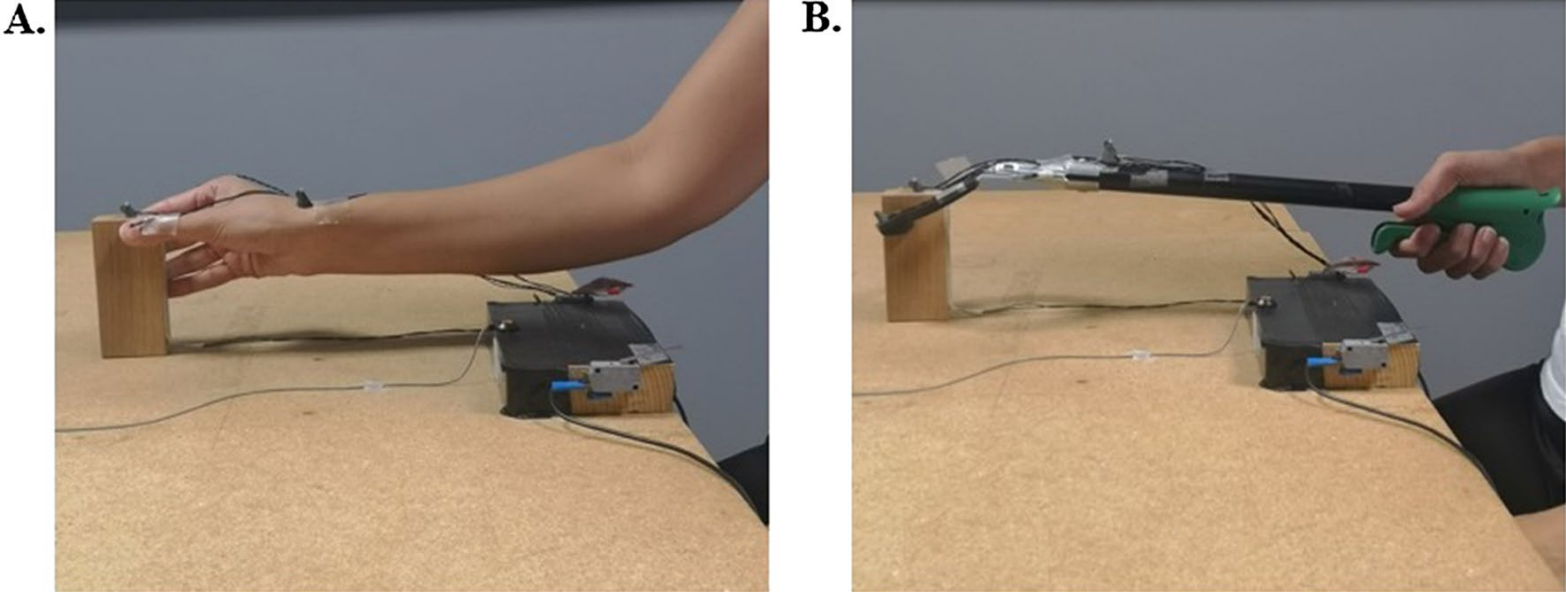
Final grasping position of a free-hand (**A**) and tool-use (**B**) reach-to-grasp movement. The tool consisted in an ergonomic handle (10 cm-long), a 30 cm-long rigid shaft and an articulated “hand” composed by two curved prongs.

The tool-use phase consisted of four blocks of 12 reach-to-grasp movements using a tool (48 trials). Participants were instructed to place the tips of the tool prongs (pinch grip) on the starting switch at the beginning of each trial and wait for an acoustic go signal to reach and grasp the object using the tool. The tool was the same used in several previous studies and consisted in an ergonomic handle (10 cm-long), a 30 cm-long rigid shaft and an articulated “hand” composed by two curved prongs with rubber allowing a stable grasp. The tool was controlled by squeezing the handle with the whole hand: closing the hand would close the tip of the prongs while opening the hand would release the grip (Fig. 2). Participants were not allowed to train with the tool, and had never used it or seen it before the trial day.

### Weighted-wrist phase

In the weighted-wrist phase, participants had to reach and grasp for the same object with their dominant hand equipped with a weight wrapped around the wrist. This task also consisted of four blocks of 12 trials each. The weight was a commercial gym wrist weight modified to equate the tool weight (300 g). As in the previous tasks, participants maintained a pinch grip position on the starting switch and waited for the acoustic go signal.

### Kinematic recordings and analysis

Three infrared light emitting diodes were taped on kinematic relevant locations on participant’s dominant hand, namely on the thumb (inside corner of the fingernail), index finger (external corner of the nail), and wrist (the skin proximal to the styloid process of the radius). The reaching component of the movement was characterized by the kinematic parameters of the wrist marker, while the grip component was characterized by the distance between the thumb and index. Three additional markers were placed on the tool in a similar functional arrangement: two on the extremities of the mechanical “fingers” and one in the distal part of the shaft “wrist”. Spatial positions of the markers were recorded with an Optotrak system (Optotrak 3020, Northern Digital Inc) placed perpendicularly over the table, with a sampling rate of 200 Hz (0.01 mm 3D resolution at 2.25 m distance). Kinematics analysis of 3D movements were performed offline to obtain for each trial the amplitude and latency of four parameters: the peaks of wrist acceleration, velocity and deceleration (transport component), as well as for the peak of grip aperture (grip component). In addition, we measured the duration of the movement.

### Statistics

#### Proprioception

A repeated measure ANOVA was done with Group (Young/Old adults) as a between subject factor, and proprioceptive test Task (IRR/IRL/CCM) and Angle (20°/40°) as a within subject factor in order to compare the proprioceptive acuity between young and old adults.

#### Kinematics

To account for the interdependency of the kinematics parameters and movement components, a full factorial design permutation analysis was applied to free-hand movements, using the flip package on R^28,29^. The factors were Session (Tool-use and Weighted-wrist) and Time (Pre- vs Post-) and their interaction to evaluate the impact of tool-use on the kinematics of movements performed before and after tool-use. Similar analyses were performed with the factors Session (Tool-use and Weighted-wrist) and Block (1st/4th) to evaluate potential learning effects of tool-use practicing. Importantly, the permutation analysis was designed for a multivariate framework. This allows to combine the significance of the kinematic parameters for the transport phase (amplitudes and latencies of peaks the wrist acceleration, velocity and deceleration) and those for the grasping phase (amplitudes and latencies of the grip aperture), to obtain one global p-value for each component. The global p-value is obtained via Nonparametric Combination (NPC^30^) of partial p-values testing the single parameters. In keeping with previous work on the same paradigm and factors^5,13^, this methodology accounts for dependence among tests through a nonparametric approach based on the joint (i.e. multivariate) permutation distribution^28,29^.

#### Length estimation

Because the forearm length estimation task involved only one parameter, a repeated measure ANOVA was performed on these data, with factors Session (Tool-use; Weighted-wrist) and Time (Pre- vs Post-session).

#### Correlations

Finally, for old participants, we performed Pearson correlations analysis between the proprioceptive tests and the delta of the kinematic changes between pre and post tool-use. The rational for this approach was to identify whether proprioceptive acuity might be linked to the ability to incorporate the tool, as assessed through kinematic changes after tool-use session.

## Results

### Online proprioception is spared in old adults

First, we compared the proprioceptive acuity between young and old adults to ascertain whether our sample of old adults displayed the expected decline in the offline (IRR/IRL) as compared to online proprioception (CCM). Noteworthy, all participants overshot the reproduced or matched target position compared to the initial reference position. The repeated measures ANOVA performed on the proprioceptive assessment revealed a significant interaction between Group and proprioceptive Task (*F*(1,37) = 3.575,* p* < 0.033). Post hoc t-tests confirmed the expected significant difference between groups for the IRR and IRL Task *(IRR error for old adults* = *5.34° vs for young adults* = *3.18°, p* < *0.001; IRL for old adults* = *5.62° vs for young adults* = *3.37°, p* < *0.001)* and the non-significant difference for the CCM Task *(CCM error for old adults* = *6.28° vs for young adults* = *5.34°, p* > *0.19)*. This pattern of results confirms the findings reported in the literature on presbypropria^15^, an alteration of older adult’s proprioception, affecting mostly the offline access to proprioceptive information^17^. Noteworthy, no significant difference was found when assessing the dominant vs non-dominant hand concerning the IR tasks (*IR for dominant hand error* = *5.57° vs for non-dominant hand error* = *5.39°, p* = *0.49*) in old adults.

### Tool-use changes movements kinematics

**Figure 3:**
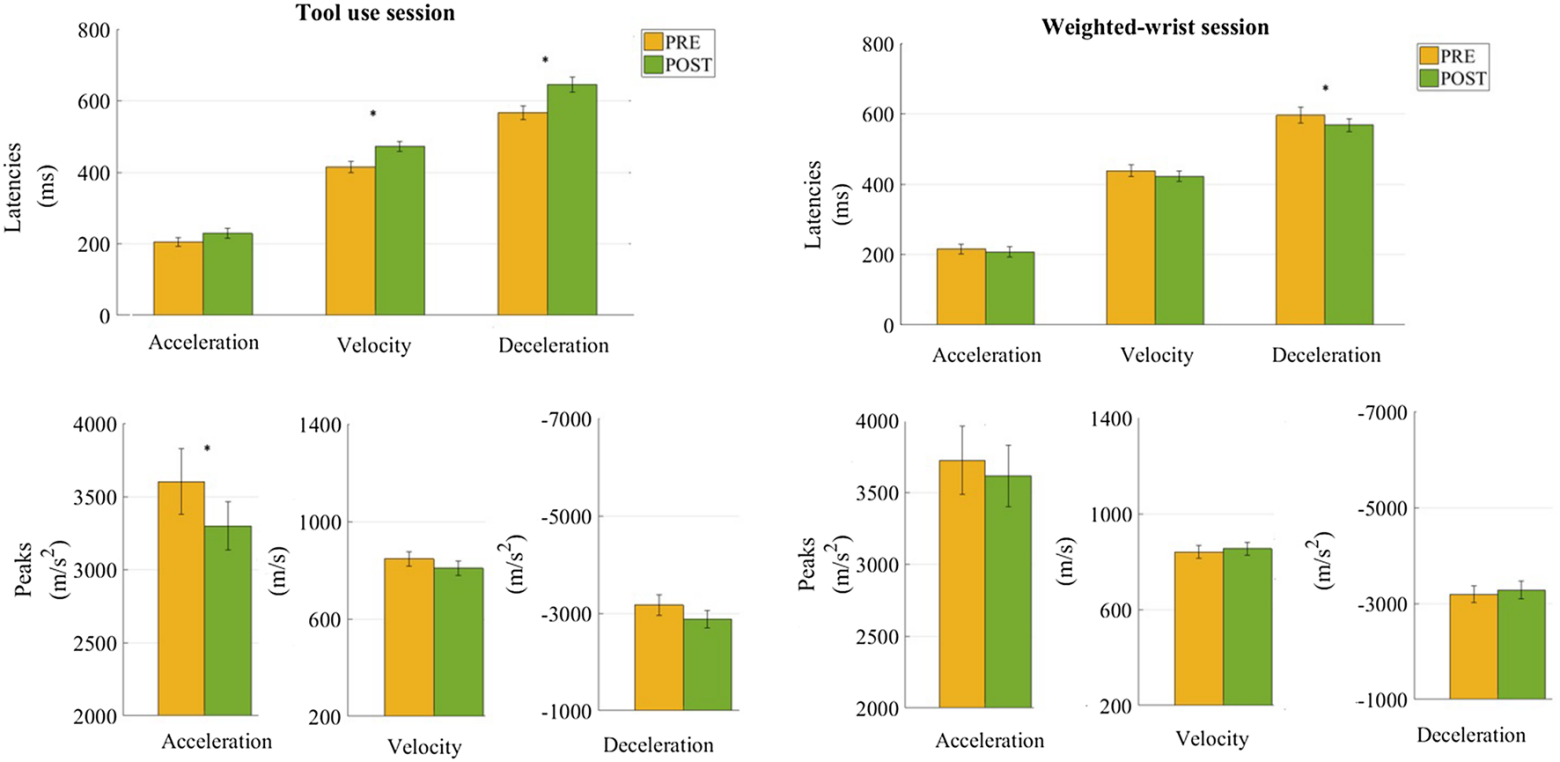
Free-hand movement kinematics modifications after tool-use (left panel) and weighted-wrist session (right panel). Bar graphs illustrate the S.E.M. Asterisks denote significant differences between pre (yellow) and post (green).

The full factorial design permutation analysis revealed that the factors Session (Tool-use and Weighted-wrist) and Time (Pre- vs Post-session) interacted significantly for the transport component *(Fisher combination; K* = *29.84, p* = *0.0007)* and the grip component *(K* = *5.95, p* < *0.03)*. The combined p-value assessed for the tool-use session confirmed an effect of Time on the transport *(K* = *31.16, p* < *0.0004)* and grip component (*K* = *8.14, p* < *0.0058).* Figure 3 shows permutations analysis performed on each parameter, which highlighted a decreased acceleration *(t* = *− 2.11, p* < *0.04)* and a delayed velocity *(t* = *4.46, p* < *0.0001)* and deceleration *(t* = *4.08, p* < *0.0001)* peaks, as well as a trend to a delayed acceleration peak and decreased velocity and deceleration peaks (*p values* < *0.068, 0.069, 0.057* respectively) after the use of the tool. In addition, the latency of the maximum grip aperture *(*pre = 622.65 ms vs post = 707.43 ms; *t* = *3.76, p* < *0.0005),* as well as the movement duration (pre = 922.96 ms vs post = 1036.68 ms; *t* = *3.43, p* < *0.0008),* were longer after the tool-use session. In sharp contrast, the analyses performed for the weighted-wrist control session yielded no significant effect of Time either on the Transport component, or on the Grip component *(both p* >  = *0.1)*.

The forearm length estimation, as assessed via the explicit length reproduction task, was not affected by the factors Session *(Tool-use and Weighted-wrist, F(1,19)* = *0.002, p* = *0.961)* and Time *(Pre- vs Post-, F(1,19)* = *0.908, p* = *353),* nor by their interactions *(average pre-tool session* = *105.48% vs average post-tool session* = *108.77% and average pre-weight session* = *106.45 vs average post-weight session* = *108.76%, F(1,19)* = *0.151, p* = *0.702).*

### Kinematics does not change throughout the course of tool-use

The full factorial design permutation analysis with factors Block (1st/4th) * Session (Tool-use and Weighted-wrist) revealed neither significant main effects nor interaction on the transport component *(Fisher combination; K* = *7.15, p* < *0.28)* or the grip component *(K* = *3.46, p* < *0.15),* ruling out any gross learning effect during sessions for this component. The grip component was affected during the use of the tool (*k* = *5.11, p* < *0.02*), as the maximum grip aperture decreased significantly from the first to the fourth block of trials (pre = 114.58 mm vs post = 106.71 mm; t = -2.69; p < 0.006).

### Online proprioceptive acuity is correlated with kinematic changes

**Figure 4:**
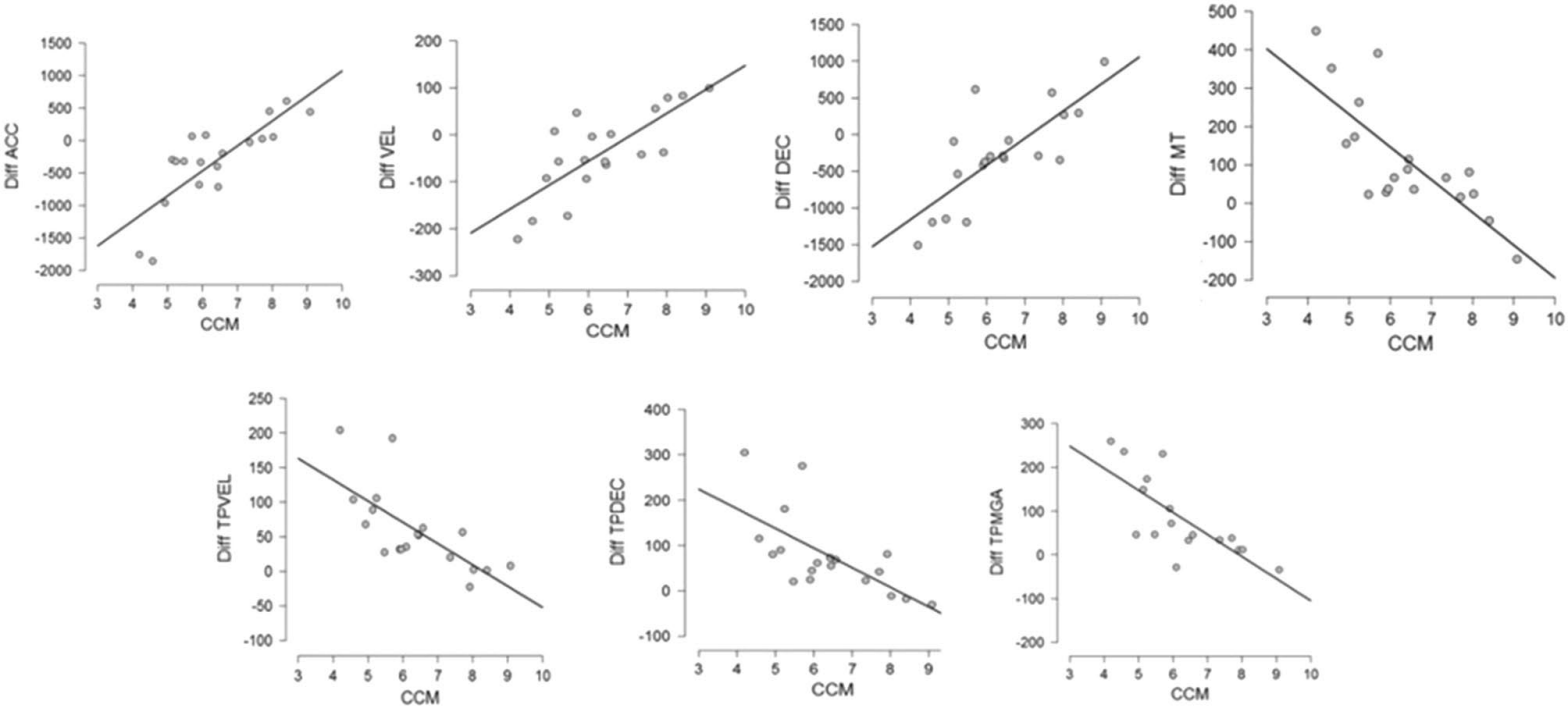
Correlation plots between error in CCM (Contralateral concurrent matching task) and the changes in kinematics induced by the tool-use session. Post minus pre difference in the tool-use session (Diff) are presented for each significant parameter (after Bonferroni correction). TP indicates the latencies to peaks. ACC, VEL and DEC denote the acceleration, velocity and deceleration peaks, respectively. MT denotes the total movement time and MGA the maximum grip aperture.

To examine the potential link between proprioception acuity and the pattern of kinematic modifications observed after tool-use, we performed in our old population a series of Pearson correlations between the absolute errors participants made in the proprioceptive tests and the kinematic changes after tool-use (post–pre difference for each parameter). There was a highly significant correlation between the online proprioceptive acuity, as measured by the CCM task, and the difference between post minus pre tool-use on most of the kinematic parameters: acceleration: r = 0.802 , p < 0.001; velocity: r = 0.769, p < 0.00012; deceleration: r = 0.766, p < 0.00013; latency of velocity: r = − 0.707, p < 0.00071; latency of deceleration: r = − 0.665, p < 0.0019; movement time: r = − 0.770, p < 0.00012; latency of maximum grip aperture: r = − 0.742, p < 0.00065 (all p values are Bonferroni corrected). As shown in Fig. 4, the better acuity in online proprioception, the larger the changes in kinematics parameters after tool-use. In striking contrast, there was no significant correlation between offline proprioceptive Task (IRR and IRL) and the pattern of kinematic changes after tool-use (all p-values > 0.12).

## Discussion

In the present study, we sought to disentangle whether online or offline access to proprioception updates the body estimate for movement execution following tool-use. To this aim, we first identified a group of ‘old’ healthy adults (60 year-old) showing declined offline, but preserved online proprioception. We then assessed them for the emergence of the pattern of kinematic changes that has been previously reported following tool-use in young adults (average 25 years-old in 7 previous studies). The results showed that relatively old participants display the typical (i.e., previously reported in young adults) kinematic signature of tool incorporation. After tool-use, the kinematics of their free-hand reaching movement’s changes, showing delayed latencies and lowered peaks amplitudes. This finding extends to the beginning of the old age the notion that tool-use modifies the unconscious estimate of the bodily effector used to control motor actions. Instead, tool-use does not modify the conscious perception of old peoples’ body, again in keeping with what known from the young population^13^. Most importantly, we found that online (but not offline) proprioceptive acuity correlated with the amount of the kinematics changes observed after tool-use, thus establishing for the first time a tight link between tool-use incorporation and real-time proprioceptive body state estimate. Altogether, these findings point to the prominent role played by online proprioception in updating the body estimate for the motor control of tools in old participants.

Previous work on the assessment of proprioception acuity mainly relied on tasks that vary in terms of either memory requirements (offline) or interhemispheric transfer (online)^17^. Our findings confirm the presence of a specific offline impairment in the old adult population, indexed by the ipsilateral matching task, as well as a preservation of online proprioceptive acuity, derived from concurrent contralateral matching task. The presence (and the nature) of proprioceptive decline with age is still debated, as is its potential contribution to motor control^31^. Its presence may vary depending on what variables are measured and which tasks are used, as is typically not observed when assessed by visual to proprioceptive matching tasks^32^. Yet, proprioceptive decline with age has been consistently reported when testing proprioceptive memory: the pattern we report here conforms to previous findings on the decline of the offline component of proprioception in healthy ageing^15,17,32^. By using the same offline/online testing procedures employed by Adamo et al.^17^, here we actually extend their results to the very beginning of the old age. While Adamo et al.’s study observed significant decline in offline proprioception in adults aged 75/76 on average^17,33^, here we report that significant decline in offline proprioception can be detected in adults aged 60 on average.

Most interesting for the scope of our study, following tool-use, old participants exhibited the typical kinematic signatures so far reported in young adults only. After tool-use, free hand reaching movements displayed protracted and lowered transport component parameters (three out of six parameters were significantly altered). Importantly, and also in line with previous work, these effects emerged specifically following tool-use. The weighted-wrist control session did not trigger any of such effects on movement’s kinematics. These tool specific kinematic modifications have been interpreted as a kinematic signature of tool incorporation in the effector representation, which would be represented as longer after tool-use^6,11^. Indeed, similar kinematics characteristics (longer latencies, reduced amplitudes) are also observable in participants having a naturally longer arm when compared to shorter-armed participants’ movements^6,13^. Finally, tool-use did not influence the conscious arm representation of old adults, as evaluated through the forearm length estimation task, as it was not affected by tool-use. Here, we further confirm that the explicit, verbal and conscious representation of the body, referred as the Body Image^34,35^, is immune to factors affecting the body representation for action^13,27^. In agreement with our predictions, these findings clearly indicate that offline proprioception is not crucial to motor control plasticity following tool-use. Would that be the case, we should expect old participants with impaired offline proprioception not to display significant changes in their movement kinematics, or displaying changes that differ from the pattern repeatedly reported in young adults. Instead, their presence and the fact that both peak amplitudes and latencies were qualitatively affected in the same directions as in the young population, suggest that preserved online proprioception plays a critical role in updating our body state estimate.

Among the first conceptualizations of unconscious body representations devoted to action control, originally called body schema, stemmed from neuropsychological observations of patients with various somatosensory diseases^36^. Since then, proprioception has been considered among the main inputs feeding this implicit body representation^37–40^. In previous work, we reported that proprioceptive deafferentation may still allow fairly accurate touch localization on a tool^41^, but prevents tool-use from affecting the subsequent kinematics^14^. This inability to incorporate a tool at motor level, at odds with what typically shown by young and now old healthy participants, attests to the crucial role of proprioceptive inputs in building an updated representation of the motor effector. Martel and coworkers^13^, by observing the same kinematic signature of tool incorporation in healthy blindfolded participants, further showed that proprioception is not only necessary, but also sufficient to trigger tool incorporation. The present results allow to step further and reveal the correlation existing between online proprioceptive acuity and the kinematics signature of the tool incorporation: the better proprioceptive acuity, the larger the extent of kinematic changes following tool-use. While these findings are in keeping with the role of proprioception in updating body representation^37,42,43^, they concur in indicating this body representation feeds internal models of motor control^1,13,14^. Our findings refine current models by suggesting that the state estimation of the body relies heavily on online proprioceptive inputs to monitor and update the represented metric of effectors. While the neural correlates of this update remain to be elucidated, the sensorimotor regions, as well as more posterior parietal cortices, could be likely candidates. Previous neuroimaging work indeed pointed to the intraparietal sulcus and the premotor cortex as neural correlate of the body estimate, and have more recently underlined the role of the somatosensory cortices in the coding of proprioception^44,45^ and position sense^46,47^.

Here we report for the first time that having used a tool that lengthens participants’ arm affects the arm length representation in the old population. The presence of these plastic changes in old adults is in keeping with the suggestion that motor control may remain relatively preserved with age, as long as it concerns relatively simple movements (see, for review^48^). Noteworthy, when comparing the kinematics during the tool-use sessions across blocks, we did not observe evidence of tool learning on the transport component. Transport parameters were comparable between the first and last blocks of trials. Importantly, this absence of gross learning effects has also been reported in previous studies^4,6,13,26^. Here however, one kinematic parameter displayed signs of tool learning, namely the tool grip aperture, whose maximum was reduced in the last compared to the first block of trials. This effect is compatible with a reduced safety margin^49^ while grasping the object as participants got used to the tool. Importantly though, after tool-use the hand maximum grip aperture did not differ from that observed before tool-use. Thus, the plastic mechanisms of tool incorporation that affect the transport component are likely independent from the processes of learning to control the tool grip aperture during tool-use.

To conclude, here we show that 60 years-old participants display plastic changes in movement kinematics of their arm following tool-use, akin to those known in young adults. As suggested elsewhere^13,14^ these changes may reflect the update of ‘state of affairs’ of the body, in particular of the effector used. Our findings clearly indicate that in old participants, the online readout of proprioceptive information provides a major contribution to this update. In addition, online proprioceptive acuity predicts the extent to which tool-use will impact subsequent freehand kinematics, corroborating the notion that tool incorporation relies heavily on ongoing processing of position sense.
